# Effects of toxic apolipoprotein E fragments on Tau phosphorylation and cognitive impairment in neonatal mice under sevoflurane anesthesia

**DOI:** 10.1002/brb3.2702

**Published:** 2022-07-10

**Authors:** Yang Yu, Man Yang, Xiaoli Zhuang, Jiacheng Pan, Yue Zhao, Yonghao Yu

**Affiliations:** ^1^ Department of Anesthesiology Tianjin Medical University General Hospital Tianjin China; ^2^ Tianjin Institute of Anesthesiology Tianjin China; ^3^ Department of Anesthesiology Sichuan University West China Hospital Chengdu China

**Keywords:** anesthesia, ApoE, ApoE fragments, ApoE‐knockout mice, ApoE‐targeted replacement mice, neurocognition, Tau phosphorylation

## Abstract

**Background:**

Anesthesia induces Tau phosphorylation and cognitive impairment in young, but not adult, mice. Apolipoprotein E (ApoE) may play a protective role in neuronal activity and injury repair, whereas its toxic fragments are reported to induce neurodegeneration and neurocognitive impairment in patients with Alzheimer's disease (AD). Therefore, we set out to test the hypothesis that the difference in ApoE fragments, but not the full‐length ApoE, contributes to the difference in Tau phosphorylation and neurocognitive functions following sevoflurane anesthesia in young mice.

**Methods:**

Sevoflurane was administered to wild‐type (WT), ApoE‐knockout (ApoE‐KO), ApoE3‐targeted replacement (ApoE3 expresses both full‐length and fragmented ApoE), and ApoE2‐targeted replacement (ApoE2 only expresses full‐length ApoE) mice. The mRNA and protein levels of ApoE, phosphorylated Tau (pTau), and cognitive function were tested in the mice.

**Results:**

Sevoflurane anesthesia enhanced ApoE mRNA, total ApoE, full‐length ApoE, ApoE fragments, Tau phosphorylation (AT8 and PHF1), and cognitive impairment in young mice, but not in adult mice. ApoE2, but not ApoE3 or ApoE‐KO, mice showed reduced sevoflurane‐induced pTau elevation and cognitive impairment.

**Conclusion:**

These data suggest that elevated ApoE fragments rather than full‐length ApoE might be one of the underlying mechanisms of age‐dependent Tau phosphorylation and cognitive impairment in young mice following sevoflurane anesthesia.

## 1 INTRODUCTION

With the fast advancement of anesthetic technology, millions of newborns and children throughout the world are undergoing surgery under general anesthesia, making child safety a major public health concern (Rappaport et al., 2011). Several clinical investigations have found that infants who have been exposed to anesthesia and surgery more than three times are more likely to acquire learning disabilities (DiMaggio et al., 2009; Flick et al., 2011; Wilder et al., 2009), while contradictory reports also exist (Davidson et al., 2016; McCann et al., 2019).

Tau is an axonal microtubule‐associated protein with the well‐known function of promoting microtubulin polymerization into microtubules, stabilizing the microtubular structure, and maintaining neuronal function (Albayram et al., 2016). It has also been proposed to play a role in dementia and cognitive impairment in Alzheimer's disease (AD) (Albayram et al., 2016; Amniai et al., 2009). Hyperphosphorylation of Tau protein is thought to be the source of AD neuropathy and cognitive impairment (Ando et al., 2016). In previous studies, we found that anesthesia with 3% sevoflurane administered twice daily for three days could cause neurotoxicity and cognitive impairment in neonatal mice (starting sevoflurane anesthesia on postnatal day 6 [P6]) but not in adult mice (starting sevoflurane anesthesia on postnatal day 60 [P60]), implying that abnormal Tau phosphorylation in the developing brain could be a key cause of anesthesia‐induced cognitive impairment (Yang et al., 2020; Yu et al., 2020). However, the mechanism through which sevoflurane‐induced Tau phosphorylation causes cognitive impairment in neonatal mice is unclear.

Apolipoprotein E (ApoE) is a component of lipoproteins that regulates lipoprotein metabolism and the cholesterol balance (Fuentes et al., 2018). ApoE is found in humans in three different alleles, ApoE2, ApoE3, and ApoE4 (Serrano‐Pozo et al., 2021). The difference between the three ApoE alleles is only two nucleotides, resulting in a protein change of two amino acids at positions 112 and 158, which leads to differences in protein stability; ApoE4 is the most unstable, followed by ApoE3, and ApoE2 is the most stable (Morrow et al., 2000; Rohn et al., 2012). Moreover, ApoE3 and ApoE4 are degraded by proteases to produce short fragments in the human brain (Wang et al., 2018). The formation of Aβ plaques, Tau hyperphosphorylation, NFT formation, and the onset of neurodegeneration and cognitive impairment in patients with AD have been associated with specific neurotoxic ApoE fragments, such as an 18 kDa N‐terminal fragment (Munoz et al., 2019; Rohn et al., 2012; Wang et al., 2018).

In the present study, we first examined the relationship between the enhancement of total ApoE, full‐length ApoE, and ApoE fragments and Tau phosphorylation and cognitive impairment after sevoflurane anesthesia in young (P6) and adult (P60) mice. The current study used wild‐type (WT), ApoE‐knockout (ApoE‐KO), ApoE3‐targeted replacement (ApoE3), and ApoE2‐targeted replacement (ApoE2) mice to investigate whether total ApoE, full‐length ApoE, or ApoE fragments play a role in the presence and severity of Tau phosphorylation and cognitive impairment in P6 mice under sevoflurane anesthesia.

The findings of the study supported the hypothesis that differences in ApoE fragments, but not full‐length ApoE levels, expressed in the hippocampus of mice of different ages contribute to differences in Tau phosphorylation and neurocognitive functions following sevoflurane anesthesia in young and adult mice.

## 2 MATERIALS AND METHODS

### 2.1 Mouse anesthesia and treatment

The animal protocol was approved by the Tianjin Medical University General Hospital Institutional Animal Care and Use Committee (Tianjin, China; clearance number, 2018‐X6‐12). The number of animals included in this study was reduced as much as possible. P6 and P60 female wild‐type (WT) and ApoE‐knockout (ApoE‐KO) C57BL/6J mice were obtained from the Nanjing Biomedical Research Institute of Nanjing University (Nanjing, China), and female ApoE3‐targeted replacement (ApoE3) and ApoE2‐targeted replacement (ApoE2) C57BL/6J mice were obtained from the Shanghai Model Organisms Center (Shanghai, China). All animals were housed in cages in a controlled setting (five adult mice or one adult mouse with litter per cage) with standard food and appropriate water (temperature 21–23°C, humidity 50–60%, 12:12 h light–dark cycle).

The mice were divided into two groups: control and sevoflurane anesthesia. They were administered sevoflurane or a control therapy from postnatal day (P) 6 to P8 or P60 to P62, and hippocampal tissues were harvested at P8 or P62. Various groups of mice were employed in behavioral research. These mice were administered sevoflurane or a control condition from P6 to P8 or P60 to P62, and the Morris water maze (MWM) test was conducted from P30 to P36 or P84 to P90. The mice in the sevoflurane group were anesthetized with 3% sevoflurane plus 60% oxygen twice daily for 3 days in a special sealed resin box chamber (20 cm × 15 cm × 7 cm) with an inlet and outlet, whereas the control mice received only 60% oxygen (balanced with nitrogen) at an equal rate of flow in a chamber similar to the anesthesia mice (Lu et al., 2017; Tao et al., 2014). The concentrations of sevoflurane and oxygen were continuously monitored using a variable anesthetic gas monitor (Vamos; Drager Medical AG & Co. KgaA, Germany) during sevoflurane anesthesia or control administration. The temperature of the anesthetic chamber was maintained at 37 ± 0.5°C using a warming pad placed beneath the chamber.

### 2.2 Brain tissue harvest

The mice were given sufficient time to recuperate from anesthesia. At P8 or P62, each mouse was decapitated and hippocampal tissues were collected. ELISA and western blot analyses were performed on the acquired hippocampal tissues. After freezing, the harvested hippocampal tissues were homogenized with immunoprecipitation buffer (M‐PER® Mammalian Protein Extraction Reagent, Cat#78501, Thermo Scientific, MA, USA), protease and phosphatase inhibitor cocktails (Cat#19541400, Roche, Switzerland), and phosphatase inhibitor cocktail (Cat#539131, Millipore, MA, USA). Lysates were collected and centrifuged at 12,000 rpm for 10 min.

### 2.3 Protein quantification

A bicinchoninic acid protein assay kit was used to assess the total protein content (BCA, Pierce, Iselin, NJ, USA).

### 2.4 Real‐time polymerase chain reaction (RT‐PCR)

Total RNA concentration was calculated using a spectrophotometer. In each group, the All‐in‐one First‐Strand cDNA synthesis kit (Cat#AORT‐0050, Gene Copoeia, USA) was used to convert 500 ng of mRNA into cDNA, which was subsequently amplified by PCR using Real Master Mix on the iQTM5 machine (SYBR Green). The 2‐Ct technique was used to calculate relative mRNA levels. The following primer sequences were used for several genes: ApoE, reverse primer, 5′‐CATGTCTTCCACTATTGGCTCG‐3′ and forward primer, 5′‐GACCCAGCAAATACGCCTG‐3′; GAPDH, reverse primer, 5′‐ AGGTCGGTGTGAACGGATTTG‐3′ and forward primer, 5′‐TGTAGACCATGTAGTTGAGGTCA‐3′.

### 2.5 Enzyme‐linked immunosorbent assay

A mouse ApoE ELISA kit (Cat# E‐EL‐M0135, Elabscience, TX, USA) was used to measure total ApoE expression levels in the hippocampi of mice in each group. In a nutshell, each well was filled with 100 μl of the standard or sample and incubated for 90 min at 37°C. After that, 100 μl of particular antibody was added to each cell and incubated for another 60 min at 37°C. After that, each well was filled with 100 μl of enzyme conjugate and incubated for 30 min at 37°C. Ninety microliters of the substrate solution was added after washing five times. The reaction was stopped 15 min later with 50 μl of terminating solution.

### 2.6 Western blot analysis

Full‐length ApoE, ApoE fragments, and Tau5, AT8, and PHF1 expressions in the hippocampus was detected using western blotting. Using a 4–20% double TIS polyacrylamide gel (Cat#M00655, Gen Script Biotech Corp., Nanjing, China) or a standard XT 4–12% double TIS gel (Bio‐Rad, USA), the samples were separated from SDS‐PAGE and transferred to nitrocellulose membranes (Bio‐Rad, USA). The membranes were then blocked with 5% skimmed milk powder and identified using the following primary antibodies: Tau5 (1:1000, Cat#ABN454, Millipore, USA), full‐length ApoE and ApoE fragments (1:4000, Cat#178479, Calbiochem, Germany), PHF1 (1:1000, Cat#3Ab184951, Abcam, UK), and AT8 (1:2000, Cat#MN1020, Thermo Fisher Scientific, USA). After washing at 4°C overnight, the membranes were incubated at 37°C for 1 h with goat anti‐mouse antibody (1:5000, Cat#31430, Invitrogen, USA). The nitrocellulose membranes were then scanned and photographed using Bio‐Rad image analysis equipment after being washed in TBST and dripped with ECL chemiluminescent liquid (Cat#34577, Invitrogen, USA). The ratio of the integral optical density of the target band to that of the β‐actin band represents the level of expression of the target protein. A 100% change refers to control or sevoflurane levels for comparison between the experimental settings.

### 2.7 Immunofluorescence

The animals were anesthetized with 3% sevoflurane for 5 min and perfused transcardially with PBS, followed by 4% paraformaldehyde in 0.1 M phosphate buffer at pH 7.4 for immunofluorescence of brain slices. The brain tissues of the mice were removed and stored at 4°C in paraformaldehyde. For immunostaining, 10 mm frozen slices of mouse brain hemispheres were used. The sections were incubated overnight at 4°C with anti‐ApoE (1:4000; Cat#178479, Calbiochem, Germany) and anti‐AT8 antibodies (1:1000; Cat#MN1020, Thermo Fisher Scientific, USA), followed by immunostaining with Alexa Fluor® 488 donkey anti‐goat IgG (1:500; Cat#A‐11055, Thermo Fisher Scientific, USA) and Alexa Fluor^®^ 594 goat anti‐mouse IgG (1:500; Cat#A‐11032, Thermo Fisher Scientific, USA) for 1 h at room temperature in dark. Finally, the slices were coated with 4′6‐diamidino‐2‐phenylindole, dihydrochloride (DAPI; Cat#104139, Abcam, UK) and incubated in a humidified dark room for 10 min before being observed in mounting fluid under a fluorescence microscope.

### 2.8 Morris water maze

MWM experiments were performed as described in our previous study (Yu et al., 2020). Briefly, P30 and P84 mice were examined in the MWM four times each day for 7 days (P30 to P36 and P84 to P90). Escape latency was measured daily. On the last day, the platform was disassembled and the platform crossing times were recorded. Each mouse was kept in a holding cage for 5 min after each experiment to dry before being returned to its home cage.

### 2.9 Statistical analysis

MWM escape latency was reported as mean ± standard deviation (SD) in separate groups, platform crossing times were presented as median with interquartile range, and other data as mean ± SD. The number of samples was 10 per group for behavioral studies, four per group for RT‐PCR, four or six per group for western blot analyses, four per group for ELISA, and three per group for immunohistochemistry analyses. The interaction between time and group variables (based on escape delay) between the two groups in the MWM was investigated using two‐way ANOVA with repeated evaluations. In addition, the Mann‐Whitney test was used to compare platform crossing times among all groups in the MWM. There were no missing data for MWM variables (escape latency and platform crossing time) throughout the data analysis. Finally, to compare the two groups for other biochemical data, the unpaired *t*‐test (if the values were in Gaussian distribution) or Mann–Whitney test (not‐Gaussian distribution) was applied. Note that *p* < .05 was judged statistically significant, and the double‐tail test was used for statistical significance. GraphPad Prism (version 5.0) and SPSS statistical software (version 21.0) were used for statistical analyses.

## 3 RESULTS

### 3.1 Sevoflurane anesthesia induced total ApoE, full‐length ApoE, and ApoE fragments increase, Tau phosphorylation enhancement, and cognitive impairment in young (P6), but not adult (P60) mice

**FIGURE 1:**
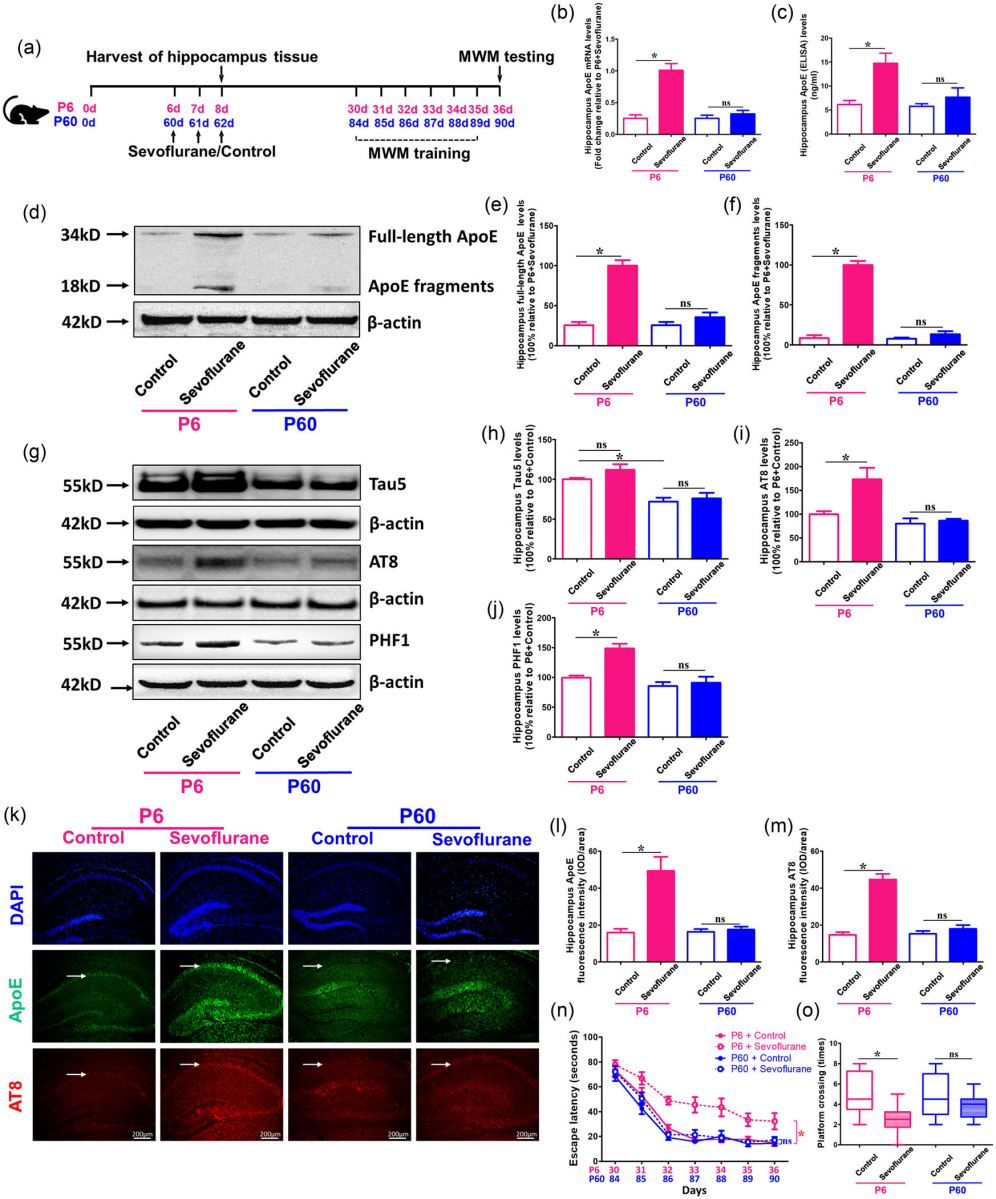
Effects of sevoflurane on ApoE and phosphorylated Tau levels as well as cognitive functions in young and adult mice. (a) Experimental design: Young (P6) and adult (P60) mice administered 3% sevoflurane + 60% O_2_ or 60% O_2_ alone for 2 h every day for 3 days (P6 to P8 and P60 to P62). In both young (P8) and adult (P62) mice, hippocampal tissues are collected at the conclusion of the sevoflurane or control condition. (b) The mRNA and (c) protein levels of total ApoE in the hippocampus of young and adult mice under the condition of sevoflurane and control are detected using RT‐PCR and ELISA, respectively (*n* = 4 mice per group). (d) The difference in the levels of full‐length ApoE and ApoE fragments in the hippocampus between young and adult mice following the sevoflurane or control condition are measured using western blot. (e, f) Summary of full‐length ApoE and ApoE fragments expression; 100% of changes correspond to P6 + Sevoflurane levels for comparison between experimental settings (*n* = 4 mice per group). (g) Western blot measurements of Tau5, AT8, and PHF1 in the hippocampus of young and adult mice following the sevoflurane or control condition. (h, i, j) Summary of Tau5, AT8, and PHF1 levels in various groups, with 100% of changes referring to P6 + Control levels for comparison between experimental circumstances (*n* = 6 mice per group). (k) Immunostaining of ApoE (green fluorescence) and AT8 (red fluorescence) expressions in the hippocampus of young and adult mice following the sevoflurane or control condition, as indicated by white arrows. (l, m) Quantification of ApoE and AT8 fluorescence intensities (*n* = 3 mice per group). (n, o) Summary of escape latency (from P30 to P36 or P84 to P90) and platform crossing (P36 or P90) in Morris Water Maze (MWM) in young and adult mice under the condition of sevoflurane or control treatment (*n* = 10 mice per group). (*p < .05)

We initially evaluated the mRNA and protein levels of total ApoE in the hippocampus of P6 and P60 animals after sevoflurane and control treatments, as shown in Figure 1(a). We observed that sevoflurane anesthesia substantially enhanced the mRNA and protein levels of total ApoE in P6 mice (*p* < .05 vs. P6 + Control) but not in P60 animals (*p* > .05 vs. P60 + Control), as compared to the control group (Figure 1b and c). Furthermore, full‐length ApoE and ApoE fragments levels were both substantially higher in the sevoflurane group compared to the control treatment only in P6 mice (*p* < .05 vs. P6 + Control), but not in P60 mice (*p* > .05 vs. P60 + Control) (Figure 1d–f).

Next, we examined the total Tau (Tau5) and phosphorylated Tau (AT8 and PHF1) levels in the hippocampus of P6 and P60 mice under sevoflurane anesthesia. The findings showed that P6 mice had greater levels of total Tau than P60 animals (*p* < .05 vs. P6 + Control). Furthermore, sevoflurane anesthesia enhanced hippocampus AT8 and PHF1 levels in P6 mice (*p* < .05 vs. P6 + Control), but not in P60 animals (*p* > .05 vs. P60 + Control) (Figure 1 g‐1j).

Finally, we investigated whether variations in ApoE and Tau phosphorylation might cause cognitive impairment in P6 and P60 animals. Immunofluorescence labeling of the hippocampus revealed that the sevoflurane group had higher expressions of ApoE and AT8 proteins than the control group in P6 mice (*p* < .05 vs. P6 + Control), but not in P60 mice (*p* > .05 vs. P60 + Control) (Figure 1k,m ). In addition, as shown in Figure 1a, we used the MWM to examine cognitive skills in young (P30 to P36) and adult mice (P84 to P90) after sevoflurane anesthesia for 7 days. We discovered that, compared to control therapy, sevoflurane anesthesia did not cause neurocognitive impairment in P60 mice (*p* > .05 vs. P60 + Control), but caused neurocognitive impairment in P6 mice (*p* < .05 vs. P6 + Control) (Figure 1n and o). These data imply that sevoflurane anesthesia increased total ApoE, full‐length ApoE, ApoE fragments, and phosphorylated Tau protein levels in the hippocampus, which might have led to neurocognitive impairment in P6 mice, but not in P60 mice.

### 3.2 ApoE deficiency could not mitigate Tau phosphorylation and cognitive impairment induced by sevoflurane anesthesia in P6 mice

**FIGURE 2:**
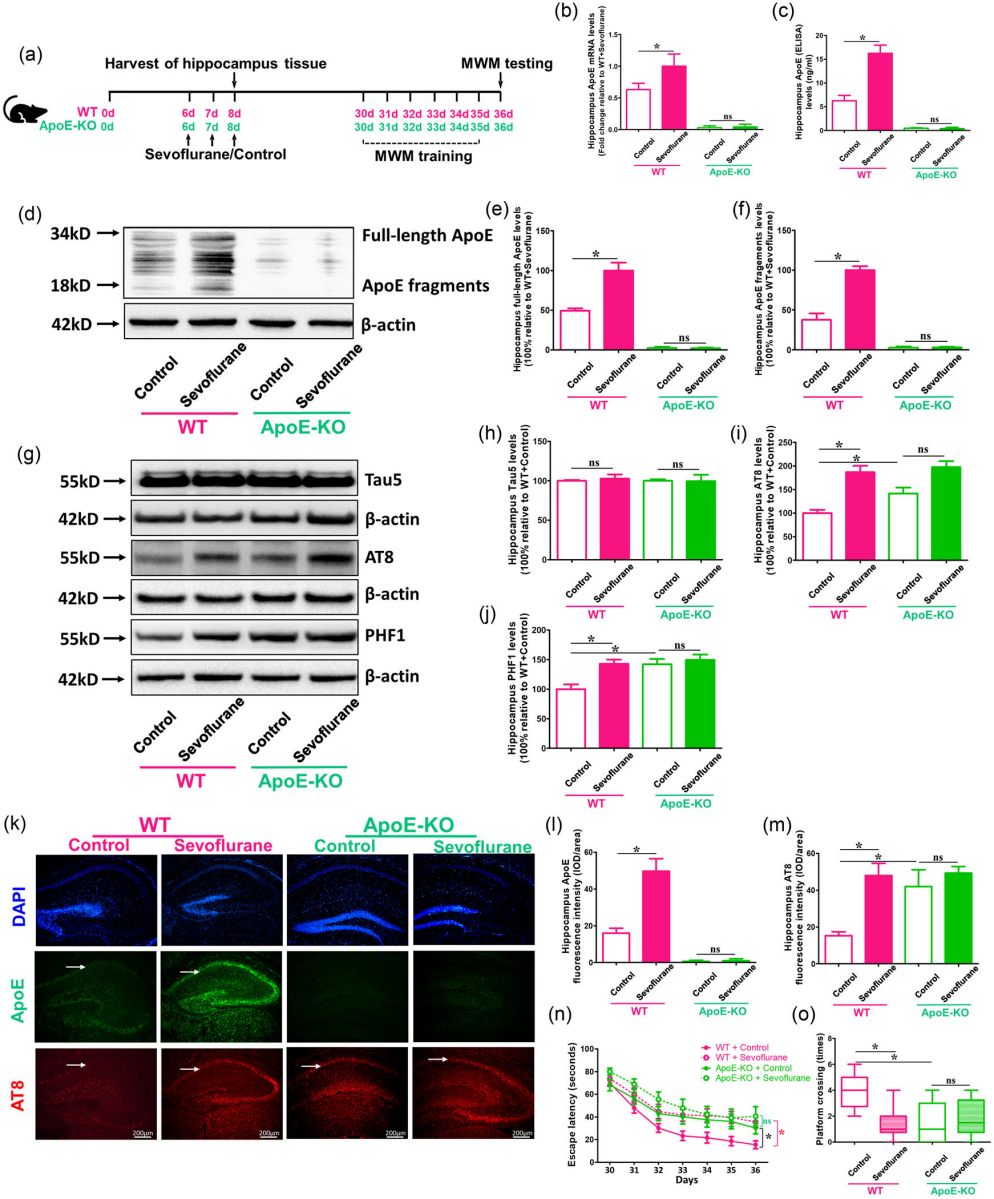
Effects of sevoflurane on ApoE and phosphorylated Tau levels and cognitive functions in WT and ApoE‐KO mice. (a) Experimental design: WT and ApoE‐KO 6‐day old mice (P6) treated with 3% sevoflurane plus 60% O_2_ or 60% O_2_ only for 2 h every day for 3 days (P6 to P8). The hippocampus tissues are harvested at the end of the sevoflurane or control condition (P8) in different groups. (b) ApoE mRNA and (c) total ApoE protein levels in the hippocampus of WT and ApoE‐KO young mice under the condition of sevoflurane and control are detected using RT‐PCR and ELISA, respectively (*n* = 4 mice per group). (d) The difference in the levels of full‐length ApoE and ApoE fragments in the hippocampus between WT and ApoE‐KO mice following the sevoflurane or control condition are measured using western blot. (e, f) Summary of full‐length ApoE and ApoE fragments expression; 100% of changes refer to WT + Sevoflurane levels for the purpose of comparison between experimental conditions (*n* = 4 mice per group). (g) Western blot measurements of Tau5, AT8, and PHF1 in the hippocampus of WT and ApoE‐KO mice following the sevoflurane or control condition. (h, i, j) Summary of Tau5, AT8. and PHF1 levels in different groups; 100% of changes refer to P6 + Control levels for the purpose of comparison between experimental conditions (*n* = 6 mice per group). (k) Immunostaining of ApoE (green fluorescence) and AT8 (red fluorescence) expressions in the hippocampus of WT and ApoE‐KO mice following the sevoflurane or control condition, as indicated by white arrows. (l, m) Quantification of ApoE and AT8 fluorescence intensities (*n* = 3 mice per group). (n, o) Summary of escape latency (from P30 to P36) and platform crossing (P36) in Morris Water Maze (MWM) in WT and ApoE‐KO mice under the condition of sevoflurane or control treatment (*n* = 10 mice per group). (*p < .05)

For further study whether ApoE could be the main reason behind sevoflurane‐induced brain damage and cognitive dysfunction in P6 mice, 6‐day old WT and ApoE‐KO mice were used in the research as shown in Figure 2(a). The results of RT‐PCR and ELISA showed that there were significant changes in total ApoE mRNA and protein levels between sevoflurane and control treatment groups in WT mice, but not in ApoE‐KO mice (*p* < .05 vs. WT + Control; *p* > .05 vs. ApoE‐KO + Control) (Figure 2b and c). In WT mice, but not ApoE‐KO, the western blot findings revealed clear differences in full‐length and fragmented ApoE expressions between sevoflurane and control therapy (*p* < .05 vs. WT + Control; *p* > .05 vs. ApoE‐KO + Control group) (Figure 2d and f). In addition, ApoE‐deficient mice had higher AT8 and PHF1 levels than WT mice (*p* < .05 vs. WT + Control), and sevoflurane treatment could induce AT8 and PHF1, but not Tau5 expression (although not statistically significant) compared with the control treatment in ApoE‐KO mice (Figure 2 g–m ). Furthermore, compared to control therapy, sevoflurane anesthesia might have exacerbated cognitive impairment in both WT and ApoE mice (*p* < .05 vs. WT + Control; *p* < .05 vs. ApoE‐KO + Control) (Figure 2n and o). These data demonstrated that ApoE could not be the therapeutic target of sevoflurane‐induced brain damage and cognitive impairment in young mice.

### 3.3 Sevoflurane‐induced phosphorylated Tau enhancement and cognitive impairment in ApoE3 mice could be mitigated in ApoE2 mice

**FIGURE 3:**
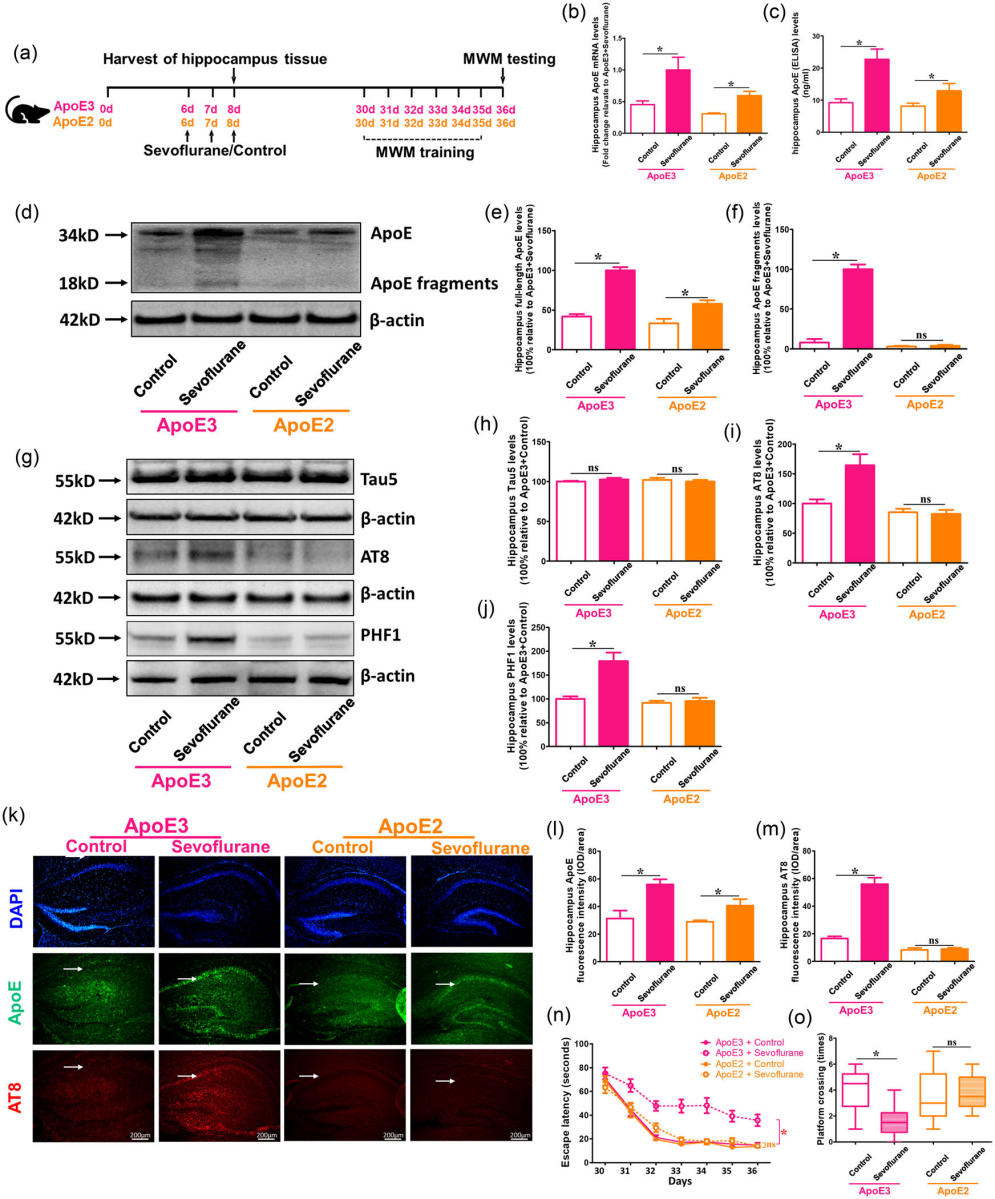
Effects of sevoflurane on ApoE and phosphorylated Tau levels and cognitive functions in ApoE3‐ and ApoE2‐target replacement mice. (a) Experimental design: ApoE3‐ and ApoE2‐target replacement young (P6) mice treated with 3% sevoflurane plus 60% O_2_ or 60% O_2_ only for 2 h every day for 3 days (P6 to P8). The hippocampus tissues are harvested at the end of the sevoflurane or control condition (P8) in different groups. (b) ApoE mRNA and (c) total ApoE protein levels in the hippocampus of ApoE3‐ and ApoE2‐target replacement young mice under the condition of sevoflurane and control are detected using RT‐PCR and ELISA, respectively (*n* = 4 mice per group). (d) The difference in the levels of full‐length ApoE and ApoE fragments in the hippocampus between ApoE3‐ and ApoE2‐target replacement young mice following the sevoflurane or control condition are measured using western blot. (e, f) Summary of full‐length ApoE and ApoE fragments expression; 100% of changes refer to WT + Sevoflurane levels for the purpose of comparison between experimental conditions (*n* = 4 mice per group). (g) Western blot measurements of Tau5, AT8, and PHF1 in the hippocampus of ApoE3‐ and ApoE2‐target replacement young mice following the sevoflurane or control condition. (h, i, j) Summary of Tau5, AT8, and PHF1 levels in different groups; 100% of changes refer to P6 + Control levels for the purpose of comparison between experimental conditions (*n* = 6 mice per group). (k) Immunostaining of ApoE (green fluorescence) and AT8 (red fluorescence) expressions in the hippocampus of WT and ApoE‐KO mice following the sevoflurane or control condition, as indicated by white arrows. (l, m) Quantification of ApoE and AT8 fluorescence intensities (*n* = 3 mice per group). (n, o) Summary of escape latency (from P30 to P36) and platform crossing (P36) in Morris Water Maze (MWM) in ApoE3‐ and ApoE2‐target replacement young mice under the condition of sevoflurane or control treatment (*n* = 10 mice per group). (*p < .05.)

To investigate whether ApoE fragments were the primary cause of sevoflurane‐induced brain injury and cognitive impairment in P6 mice, 6‐day‐old ApoE3‐ and ApoE2‐target replacement animals were used in the investigation, as shown in Figure 3(a). Sevoflurane anesthesia increased total ApoE mRNA and protein levels in both ApoE3‐ and ApoE2‐target replacement mice, according to RT‐PCR and ELISA data (*p* < .05 vs ApoE2 + Control; *p* < .05 versus ApoE2 + Control) (Figure 3b and c). The western blot findings revealed significant changes in full‐length ApoE expression between sevoflurane and control therapy in ApoE3, but not ApoE2, mice (*p* < .05 vs. WT + Control; *p* > .05 vs. ApoE‐KO + Control) (Figure 3d and f). In fact, the quantity of ApoE fragments produced by ApoE2 target‐replacement mice was low, regardless of the use of sevoflurane. Furthermore, in ApoE2 mice, sevoflurane anesthesia did not enhance AT8 and PHF1 levels compared to the control treatment (*p* > .05 vs. ApoE2 + Control); however, in ApoE3 mice, there were greater AT8 and PHF1 expressions in the sevoflurane group than in the control group (*p* < .05 vs. ApoE3 + Control) (Figure 3 g‐‐m ). Furthermore, when compared to the control group, sevoflurane anesthesia might have exacerbated cognitive impairment only in ApoE3‐target replacement mice but not in ApoE2‐target replacement mice (P 0.05 vs. ApoE3 + Control; *p* > .05 vs. ApoE2 + Control) (Figure 3n and o). These findings suggest that the fragments, but not full‐length ApoE, are the main reason for enhanced Tau phosphorylation and neurocognitive impairment following sevoflurane anesthesia in vivo.

## 4 DISCUSSION

We only chose female mice in this research since it has previously been shown that females are more vulnerable to anesthetic than males, resulting in long‐term cognitive impairment(Yang et al., 2020; Zhang et al., 2017). As a result, the mice utilized in this experiment were entirely female in order to generate a consistent cognitive impairment model. In the current study, we found that young (P6) mice had higher total Tau expression (both at baseline and after sevoflurane treatment) than adult (P60) mice, and sevoflurane anesthesia produced higher hippocampal levels of total ApoE, full‐length ApoE, ApoE fragments, and phosphorylated Tau proteins (AT8 and PHF1) in P6 mice than in P60 mice. Furthermore, repeated sevoflurane anesthesia might elicit neurocognitive impairment only in P6 mice but not in P60 animals. ApoE deficiency, on the other hand, had no effect on sevoflurane‐induced Tau phosphorylation and neurocognitive impairment. In contrast, sevoflurane anesthesia caused Tau phosphorylation and cognitive impairment in juvenile ApoE‐KO mice. Finally, missing ApoE segments other than full‐length ApoE in ApoE2‐target replacement mice reduced sevoflurane‐induced Tau phosphorylation and neurocognitive impairment relative to ApoE3‐target replacement animals. These findings show that the increase in toxic ApoE fragments after sevoflurane anesthesia in young mice, but not in older mice, leads to a difference in Tau phosphorylation and neurocognitive performance between the two groups. These findings also imply that children with ApoE2 alleles who are treated with repeated anesthetics may not have learning deficits in the future, pending further research.

Several published studies have found that hyperphosphorylated Tau proteins in the brain play important roles in anesthesia‐induced cognitive impairment in young mice (Shen et al., 2013; Tao et al., 2014; Vutskits & Xie, 2016), but the underlying mechanisms, particularly the upstream mechanism by which anesthesia‐induced Tau phosphorylation leads to cognitive impairment in young mice, are still unknown. ApoE is a 35‐kDa glycoprotein that is extensively expressed in the human body and serves as a lipid transporter (Munoz et al., 2019). However, several studies have suggested that these activities may go beyond lipid metabolism to impact Alzheimer's disease and other neurodegenerative disorders, such as tauopathy (Shi et al., 2017; Shinohara & Sato, 2019). ApoE has been shown to directly bind Tau protein independent of Aβ in vitro, and ApoE neuronal expression in vivo leads to Tau hyperphosphorylation (Brecht et al., 2004; Shi et al., 2017; Strittmatter et al., 1994). In the current study, we discovered that repeated sevoflurane anesthesia increased hippocampal levels of total ApoE, full‐length ApoE, ApoE fragments, Tau phosphorylation (AT8 and PHF1), as well as cognitive impairment in young mice, but not in adult animals (Figure 1). These findings show that cognitive impairment mediated by sevoflurane anesthesia‐induced Tau phosphorylation may be linked to an increase in ApoE.

To further investigate whether total ApoE plays a role in the presence and degree of Tau phosphorylation and cognitive impairment in young mice under sevoflurane anesthesia, WT and ApoE‐KO mice were used in the current investigation. We discovered that a lack of total ApoE did not prevent the phosphorylation of Tau or the development of cognitive impairment caused by sevoflurane anesthesia (Figure 2). According to previous studies, ApoE can be a carrier of cholesterol essential for neuronal function and injury repair in the brain, and ApoE deficiency can induce blood‐brain barrier (BBB) impairment, cause oxidative damage to neurons, and impede synapse growth, resulting in neurodegeneration (Fuentes et al., 2018; Getz & Reardon, 2016; Liu et al., 2019). As a result, animals lacking the ApoE gene displayed cognitive impairment.

ApoE is degraded by proteins in the human brain, resulting in shortened fragments based on the stability of various genotypes (ApoE2 > ApoE3 > ApoE4). Several studies have linked certain ApoE fragments to the formation of Aβ plaques, Tau hyperphosphorylation, NFT formation, and the onset of neurodegeneration (Brecht et al., 2004; Harris et al., 2003; Shinohara & Sato, 2019). We used ApoE3 and ApoE2‐targeted replacement mice in our present investigation to examine whether full‐length ApoE or ApoE fragments played a role in the development of Tau phosphorylation and cognitive impairment in young mice under sevoflurane anesthesia. We picked ApoE3 as the “wild type” allele since it is the most frequent subtype in the population, with an allele frequency of 70–80%, and produces less hazardous fragments under normal settings than ApoE4 allele (Safieh et al., 2019; Wang et al., 2018). ApoE2, which is the most stable and least likely to split into fragments of the three alleles, was chosen as the “ApoE fragments deficient” model (Morrow et al., 2000; Rohn et al., 2012). We discovered that sevoflurane anesthesia might induce greater full‐length ApoE levels than the control condition in the hippocampus of ApoE3‐ and ApoE2‐target replacement mice. Sevoflurane therapy, on the other hand, increased the quantities of toxic fragments solely in ApoE3 animals, with no toxic fragments formed in ApoE2 mice under anesthesia or in normal setting. Furthermore, the sevoflurane‐induced increase in phosphorylated Tau and cognitive impairment in ApoE3 mice might be reduced in ApoE2 animals (Figure 3). These findings suggest that toxic ApoE fragments, rather than full‐length ApoE, may be the primary cause of Tau phosphorylation and neurocognitive impairment caused by sevoflurane anesthesia in young mice. The reason young mice have higher amounts of harmful ApoE fragments after anesthesia might be related to serine protease release, such as high temperature requirement factor 1 (HtrA1) (Chu et al., 2016; Munoz et al., 2019); however, this remains to be established in future investigations.

## 5 CONCLUSIONS

In conclusion, there may be a balance between full‐length ApoE and fragmentation of ApoE in the normal brain, with the former being the protective component and the latter being the toxic part. Under normal conditions, when detrimental stimulations are present, the brain may initially create full‐length ApoE to activate and heal itself from damage. However, if the damaging stimuli continue, the full‐length ApoE may divide into many pieces, disrupting the ApoE equilibrium in the brain and potentially leading to neurotoxicity and neurodegeneration, as well as Tau hyperphosphorylation and neurocognitive impairment. Overall, amplification of ApoE fragments, but not full‐length ApoE, might be one of the underlying mechanisms of age‐dependent Tau phosphorylation and neurocognitive impairment in young mice after sevoflurane anesthesia. These findings have led to the identification of possible targets for the prevention and treatment of postoperative neurocognitive impairment in children.

## CONFLICT OF INTEREST

The authors declare no competing interest.

### PEER REVIEW

The peer review history for this article is available at https://publons.com/publon/10.1002/brb3.2702.

## Data Availability

The datasets used and analyzed in the present research are available from the corresponding author on reasonable request.

## References

[brb32702-bib-0001] Albayram, O. , Herbert, M. K. , Kondo, A. , Tsai, C. Y. , Baxley, S. , Lian, X. , Hansen, M. , Zhou, X. Z. , & Lu, K. P. (2016). Function and regulation of tau conformations in the development and treatment of traumatic brain injury and neurodegeneration. Cell & Bioscience, 6, 59. 10.1186/s13578-016-0124-4 27980715 PMC5139118

[brb32702-bib-0002] Amniai, L. , Barbier, P. , Sillen, A. , Wieruszeski, J. M. , Peyrot, V. , Lippens, G. , & Landrieu, I. (2009). Alzheimer disease specific phosphoepitopes of Tau interfere with assembly of tubulin but not binding to microtubules. Faseb Journal, 23(4), 1146–1152. 10.1096/fj.08-121590 19074508

[brb32702-bib-0003] Ando, K. , Oka, M. , Ohtake, Y. , Hayashishita, M. , Shimizu, S. , Hisanaga, S. , & Iijima, K. M. (2016). Tau phosphorylation at Alzheimer's disease‐related Ser356 contributes to tau stabilization when PAR‐1/MARK activity is elevated. Biochemical and Biophysical Research Communications, 478(2), 929–934. 10.1016/j.bbrc.2016.08.053 27520376 PMC5675734

[brb32702-bib-0004] Brecht, W. J. , Harris, F. M. , Chang, S. , Tesseur, I. , Yu, G. Q. , Xu, Q. , Fish, J. D. , Wyss‐Coray, T. , Buttini, M. , Mucke, L. , Mahley, R. W. , & Huang, Y. (2004). Neuron‐specific apolipoprotein e4 proteolysis is associated with increased tau phosphorylation in brains of transgenic mice. Journal of Neuroscience, 24(10), 2527–2534. 10.1523/JNEUROSCI.4315-03.2004 15014128 PMC6729489

[brb32702-bib-0005] Chu, Q. , Diedrich, J. K. , Vaughan, J. M. , Donaldson, C. J. , Nunn, M. F. , Lee, K. F. , & Saghatelian, A. (2016). HtrA1 proteolysis of ApoE in vitro is allele selective. Journal of the American Chemical Society, 138(30), 9473–9478. 10.1021/jacs.6b03463 27379525 PMC5063305

[brb32702-bib-0006] Davidson, A. J. , Disma, N. , de Graaff, J. C. , Withington, D. E. , Dorris, L. , Bell, G. , Stargatt, R. , Bellinger, D. C. , Schuster, T. , Arnup, S. J. , Hardy, P. , Hunt, R. W. , Takagi, M. J. , Giribaldi, G. , Hartmann, P. L. , Salvo, I. , Morton, N. S. , von Ungern Sternberg, B. S. , & Locatelli, B. G. , G. A. S. consortium . (2016). Neurodevelopmental outcome at 2 years of age after general anaesthesia and awake‐regional anaesthesia in infancy (GAS): An international multicentre, randomised controlled trial. Lancet, 387(10015), 239–250. 10.1016/S0140-6736(15)00608-X 26507180 PMC5023520

[brb32702-bib-0007] DiMaggio, C. , Sun, L. S. , Kakavouli, A. , Byrne, M. W. , & Li, G. (2009). A retrospective cohort study of the association of anesthesia and hernia repair surgery with behavioral and developmental disorders in young children. Journal of Neurosurgical Anesthesiology, 21(4), 286–291. 10.1097/ANA.0b013e3181a71f11 19955889 PMC2789336

[brb32702-bib-0008] Flick, R. P. , Katusic, S. K. , Colligan, R. C. , Wilder, R. T. , Voigt, R. G. , Olson, M. D. , Sprung, J. , Weaver, A. L. , Schroeder, D. R. , & Warner, D. O. (2011). Cognitive and behavioral outcomes after early exposure to anesthesia and surgery. Pediatrics, 128(5), e1053–e1061. 10.1542/peds.2011-0351 21969289 PMC3307194

[brb32702-bib-0009] Fuentes, D. , Fernandez, N. , Garcia, Y. , Garcia, T. , Morales, A. R. , & Menendez, R. (2018). Age‐related changes in the behavior of apolipoprotein E knockout mice. Behavioral Sciences (Basel), 8(3), 33. 10.3390/bs8030033 PMC5867486 29510495

[brb32702-bib-0010] Getz, G. S. , & Reardon, C. A. (2016). ApoE knockout and knockin mice: The history of their contribution to the understanding of atherogenesis. Journal of Lipid Research, 57(5), 758–766. 10.1194/jlr.R067249 27015743 PMC4847636

[brb32702-bib-0011] Harris, F. M. , Brecht, W. J. , Xu, Q. , Tesseur, I. , Kekonius, L. , Wyss‐Coray, T. , & Huang, Y. (2003). Carboxyl‐terminal‐truncated apolipoprotein E4 causes Alzheimer's disease‐like neurodegeneration and behavioral deficits in transgenic mice. PNAS, 100(19), 10966–10971. 10.1073/pnas.1434398100 12939405 PMC196910

[brb32702-bib-0012] Liu, H. , Zhang, Y. , Sun, S. , & Wang, S. (2019). Efficacy of terpenoid in attenuating aortic atherosclerosis in Apolipoprotein‐E deficient mice: A meta‐analysis of animal studies. BioMed Research International, 2019, 2931831. 10.1155/2019/2931831 31392210 PMC6662500

[brb32702-bib-0013] Lu, H. , Liufu, N. , Dong, Y. , Xu, G. , Zhang, Y. , Shu, L. , …, & Xie, Z. (2017). Sevoflurane acts on ubiquitination‐proteasome pathway to reduce postsynaptic density 95 protein levels in young mice. Anesthesiology, 127(6), 961–975. 10.1097/ALN.0000000000001889 28968276 PMC5685882

[brb32702-bib-0014] McCann, M. E. , de Graaff, J. C. , Dorris, L. , Disma, N. , Withington, D. , Bell, G. , …, & Consortium, G. A. S. (2019). Neurodevelopmental outcome at 5 years of age after general anaesthesia or awake‐regional anaesthesia in infancy (GAS): An international, multicentre, randomised, controlled equivalence trial. Lancet, 393(10172), 664–677. 10.1016/S0140-6736(18)32485-1 30782342 PMC6500739

[brb32702-bib-0015] Morrow, J. A. , Segall, M. L. , Lund‐Katz, S. , Phillips, M. C. , Knapp, M. , Rupp, B. , & Weisgraber, K. H. (2000). Differences in stability among the human apolipoprotein E isoforms determined by the amino‐terminal domain. Biochemistry, 39(38), 11657–11666. 10.1021/bi000099m 10995233

[brb32702-bib-0016] Munoz, S. S. , Garner, B. , & Ooi, L. (2019). Understanding the role of ApoE fragments in alzheimer's disease. Neurochemical Research, 44(6), 1297–1305. 10.1007/s11064-018-2629-1 30225748

[brb32702-bib-0017] Rappaport, B. , Mellon, R. D. , Simone, A. , & Woodcock, J. (2011). Defining safe use of anesthesia in children. New England Journal of Medicine, 364(15), 1387–1390. 10.1056/NEJMp1102155 21388302

[brb32702-bib-0018] Rohn, T. T. , Catlin, L. W. , Coonse, K. G. , & Habig, J. W. (2012). Identification of an amino‐terminal fragment of apolipoprotein E4 that localizes to neurofibrillary tangles of the Alzheimer's disease brain. Brain Research, 1475, 106–115. 10.1016/j.brainres.2012.08.003 22902767

[brb32702-bib-0019] Safieh, M. , Korczyn, A. D. , & Michaelson, D. M. (2019). ApoE4: An emerging therapeutic target for Alzheimer's disease. Bmc Medicine [Electronic Resource], 17(1), 64. 10.1186/s12916-019-1299-4 PMC6425600 30890171

[brb32702-bib-0020] Serrano‐Pozo, A. , Das, S. , & Hyman, B. T. (2021). APOE and Alzheimer's disease: Advances in genetics, pathophysiology, and therapeutic approaches. Lancet Neurology, 20(1), 68–80. 10.1016/S1474-4422(20)30412-9 33340485 PMC8096522

[brb32702-bib-0021] Shen, X. , Dong, Y. , Xu, Z. , Wang, H. , Miao, C. , Soriano, S. G. , …, & Xie, Z. (2013). Selective anesthesia‐induced neuroinflammation in developing mouse brain and cognitive impairment. Anesthesiology, 118(3), 502–515. 10.1097/ALN.0b013e3182834d77 23314110 PMC3580002

[brb32702-bib-0022] Shi, Y. , Yamada, K. , Liddelow, S. A. , Smith, S. T. , Zhao, L. , Luo, W. , …, & Holtzman, D. M. (2017). ApoE4 markedly exacerbates tau‐mediated neurodegeneration in a mouse model of tauopathy. Nature, 549(7673), 523–527. 10.1038/nature24016 28959956 PMC5641217

[brb32702-bib-0023] Shinohara, M. , & Sato, N. (2019). The roles of apolipoprotein E, lipids, and glucose in the pathogenesis of Alzheimer's disease. Advances in Experimental Medicine and Biology, 1128, 85–101. 10.1007/978-981-13-3540-2_5 31062326

[brb32702-bib-0024] Strittmatter, W. J. , Saunders, A. M. , Goedert, M. , Weisgraber, K. H. , Dong, L. M. , Jakes, R. , …, & Roses, A. D. (1994). Isoform‐specific interactions of apolipoprotein E with microtubule‐associated protein tau: Implications for Alzheimer disease. Proceedings of the National Academy of Sciences of the United States of America, 91(23), 11183–11186. 10.1073/pnas.91.23.11183 7972031 PMC45191

[brb32702-bib-0025] Tao, G. , Zhang, J. , Zhang, L. , Dong, Y. , Yu, B. , Crosby, G. , …, & Xie, Z. (2014). Sevoflurane induces tau phosphorylation and glycogen synthase kinase 3beta activation in young mice. Anesthesiology, 121(3), 510–527. 10.1097/ALN.0000000000000278 24787352 PMC4165789

[brb32702-bib-0026] Vutskits, L. , & Xie, Z. (2016). Lasting impact of general anaesthesia on the brain: Mechanisms and relevance. Nature Reviews Neuroscience, 17(11), 705–717. 10.1038/nrn.2016.128 27752068

[brb32702-bib-0027] Wang, C. , Najm, R. , Xu, Q. , Jeong, D. E. , Walker, D. , Balestra, M. E. , …, & Huang, Y. (2018). Gain of toxic apolipoprotein E4 effects in human iPSC‐derived neurons is ameliorated by a small‐molecule structure corrector. Nature Medicine, 24(5), 647–657. 10.1038/s41591-018-0004-z PMC5948154 29632371

[brb32702-bib-0028] Wilder, R. T. , Flick, R. P. , Sprung, J. , Katusic, S. K. , Barbaresi, W. J. , Mickelson, C. , …, & Warner, D. O. (2009). Early exposure to anesthesia and learning disabilities in a population‐based birth cohort. Anesthesiology, 110(4), 796–804. 10.1097/01.anes.0000344728.34332.5d 19293700 PMC2729550

[brb32702-bib-0029] Yang, M. , Tan, H. , Zhang, K. , Lian, N. , Yu, Y. , & Yu, Y. (2020). Protective effects of Coenzyme Q10 against sevoflurane‐induced cognitive impairment through regulating apolipoprotein E and phosphorylated Tau expression in young mice. International Journal of Developmental Neuroscience, 10.1002/jdn.10041 32473608

[brb32702-bib-0030] Yu, Y. , Yang, Y. , Tan, H. , Boukhali, M. , Khatri, A. , Yu, Y. , …, & Xie, Z. (2020). Tau contributes to sevoflurane‐induced neurocognitive impairment in neonatal mice. Anesthesiology, 133(3), 595–610. 10.1097/ALN.0000000000003452 32701572 PMC7429299

[brb32702-bib-0031] Zhang, C. , Zhang, Y. , Shen, Y. , Zhao, G. , Xie, Z. , & Dong, Y. (2017). Anesthesia/surgery induces cognitive impairment in female alzheimer's disease transgenic mice. Journal of Alzheimer's Disease, 57(2), 505–518. 10.3233/JAD-161268 28269788

